# Development and characterization of microsatellite markers for *Morus spp*. and assessment of their transferability to other closely related species

**DOI:** 10.1186/1471-2229-13-194

**Published:** 2013-12-01

**Authors:** Balachandran Mathithumilan, Niteen Narharirao Kadam, Jyoti Biradar, Sowmya H Reddy, Mahadeva Ankaiah, Madhura J Narayanan, Udayakumar Makarla, Paramjit Khurana, Sheshshayee Madavalam Sreeman

**Affiliations:** 1 Department of Crop Physiology, University of Agricultural Sciences, Bangalore, India; 2 Department of Sericulture, University of Agricultural Sciences, Bangalore, India; 3 Department of Plant Molecular Biology, University of Delhi, South Campus, New Delhi, India

## Abstract

**Background:**

Adoption of genomics based breeding has emerged as a promising approach for achieving comprehensive crop improvement. Such an approach is more relevant in the case of perennial species like mulberry. However, unavailability of genomic resources of co-dominant marker systems has been the major constraint for adopting molecular breeding to achieve genetic enhancement of Mulberry. The goal of this study was to develop and characterize a large number of locus specific genic and genomic SSR markers which can be effectively used for molecular characterization of mulberry species/genotypes.

**Result:**

We analyzed a total of 3485 DNA sequences including genomic and expressed sequences (ESTs) of mulberry (*Morus alba* L.) genome. We identified 358 sequences to develop appropriate microsatellite primer pairs representing 222 genomic and 136 EST regions. Primers amplifying locus specific regions of Dudia white (a genotype of *Morus alba* L), were identified and 137 genomic and 51 genic SSR markers were standardized. A two pronged strategy was adopted to assess the applicability of these SSR markers using mulberry species and genotypes along with a few closely related species belonging to the family *Moraceae* viz., Ficus, Fig and Jackfruit. While 100% of these markers amplified specific loci on the mulberry genome, 79% were transferable to other related species indicating the robustness of these markers and the potential they hold in analyzing the molecular and genetic diversity among mulberry germplasm as well as other related species. The inherent ability of these markers in detecting heterozygosity combined with a high average polymorphic information content (PIC) of 0.559 ranging between 0.076 and 0.943 clearly demonstrates their potential as genomic resources in diversity analysis. The dissimilarity coefficient determined based on Neighbor joining method, revealed that the markers were successful in segregating the mulberry species, genotypes and other related species into distinct clusters.

**Conclusion:**

We report a total of 188 genomic and genic SSR markers in *Morus alba* L. A large proportion of these markers (164) were polymorphic both among mulberry species and genotypes. A substantial number of these markers (149) were also transferable to other related species like Ficus, Fig and Jackfruit. The extent of polymorphism revealed and the ability to detect heterozygosity among the cross pollinated mulberry species and genotypes render these markers an invaluable genomic resource that can be utilized in assessing molecular diversity as well as in QTL mapping and subsequently mulberry crop improvement through MAS.

## Background

Mulberry, a perennial out-breeding tree species is distributed in varied environments ranging from tropical to sub-arctic regions. The wide distribution can be attributed to its capability to adapt to diverse agro-climatic conditions, fast regeneration and both sexual and asexual modes of propagation. The mulberry leaf serves as the sole source of food to the domesticated silkworm, *Bombyxmori* L., and hence contributes significantly to the success of silk industry in India. It is predicted that around 27,000 MT of raw silk would need to be produced by the year 2030 to meet the demand in India [1]. This goal is strongly dependant on improving mulberry productivity. Enhancing the yield potential and minimizing the yield loss due to stresses are therefore the most viable strategies to achieve genetic enhancement of mulberry [2].

Despite the significant progress achieved so far, genetic improvement of mulberry yield potential through conventional breeding has been distressingly slow, mainly because of the perennial growth habit and complex inheritance pattern. Convincing evidences suggest that relevant traits need to be introgressed onto an elite genetic background to achieve greater success in crop improvement endeavors. Thus, the applications of modern molecular and genomic tools are expected to strongly complement the breeding efforts in enhancing yield potential of mulberry [2]. Advances in PCR based genomic approaches have generated robust DNA marker systems [3,4], which offer an effective approach to augment breeding methods for mulberry improvement [5]. Randomly amplified polymorphic DNA (RAPD), Amplified fragment length polymorphism (AFLP) and Inter simple sequence repeats (ISSR) have been the most frequently employed marker systems to study the genetic diversity among mulberry species and genotypes [6-8]. Though these marker systems provide a good option to discriminate the evolutionary relationships among species [9], being dominant, RAPD, AFLP and ISSR markers have limited application in marker assisted breeding, especially in heterozygous out-breeding perennial species like mulberry. Lack of sufficient number of co-dominant marker systems renders molecular breeding practices in mulberry still a distant possibility.

Microsatellites or simple sequence repeats (SSR) are short stretches of tandemly repeated DNA sequences, distributed throughout the eukaryotic genome [10,11]. SSR markers display locus specificity, are co-dominant and highly transferable to other related species [12] and hence are the most attractive choice of marker systems for mulberry. Further, the higher ability to detect polymorphism by the SSR markers is an added advantage while analyzing closely related species and/or genotypes, which is often the case in breeding programs [13]. The efficiency of the SSR markers in genetic screening has been reported in tree species like peach, olive and fig [14-16].

Except for the reports of Aggarwal et al. [17] and Zhao et al. [18], there have not been many efforts in developing co-dominant markers in mulberry. From this background, the main aim of this work was to generate SSR markers for characterizing mulberry germplasm and/or mapping populations. We report a large number of genic and genomic SSR markers for mulberry and examined their transferability to closely related species like Ficus (*Ficusbengalensis*), Fig (*Ficuscarica*) and Jackfruit (*Artocarpusheterophyllus*).

## Result and discussion

Pre-cloning enrichment strategy was adopted to isolate the genomic microsatellite regions and a set of previously characterized expressed sequence tags (ESTs) [19-21] were analyzed to identify genic microsatellite regions. A total of 3485 sequences, including 1094 genomic and 2391 EST sequences were analyzed for the presence of microsatellite regions. Locus specific primers were designed for such target sequences to develop SSR markers.

### Isolation and characterization of genomic microsatellites

**Figure 1:**
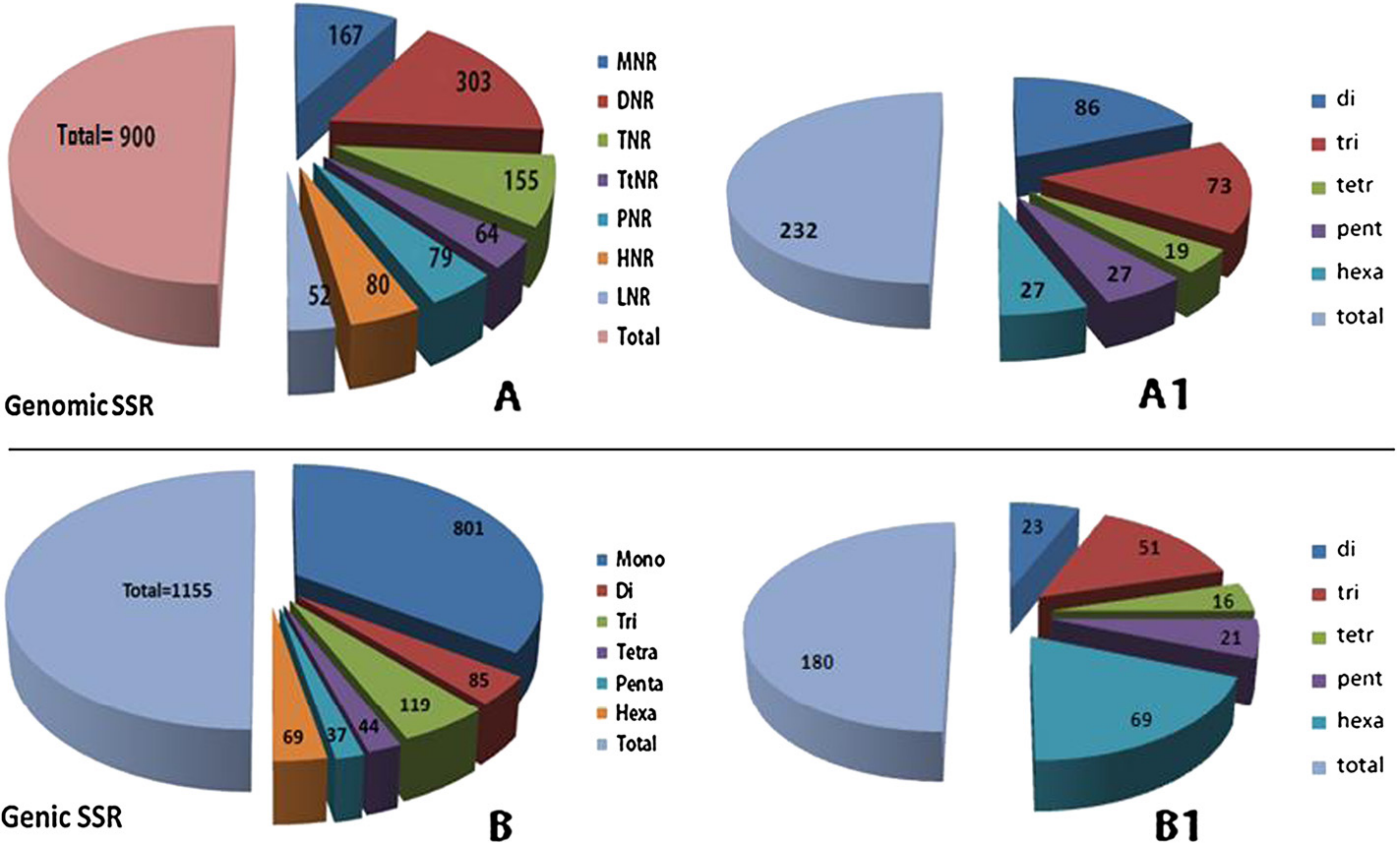
**Classification and diversity of repeat types among the identified genomic and genic microsatellite motifs.** The total number of microsatellite motifs on genomic sequences is illustrated in panel **A** while the genic microsatellites are in **B**. The locus specific marker diversity of genomic and genic microsatellites is illustrated in **A1** and **B1**, respectively.

Analysis of the genomic sequences revealed a total of 900 diverse microsatellite loci (Table 1). Among them, 167 (18.56%) sequences had mono nucleotide repeats (MNR) followed by 303 (33.67%) sequences with di-nucleotide repeats (DNR). Tri nucleotide repeats (TNR) were found among 155 (17.22%) sequences while tetra (TtNR), penta (PNR) and hexa (HNR) nucleotide repeats were relatively less frequent in the enrichment library (Figure 1). Besides these types, 52 (5.78%) microsatellite loci with repeat motifs having more than six nucleotide bases referred to as long nucleotide repeats (LNR) were also identified. It is well accepted that di, tri, tetra, penta and hexa repeat motifs represent an appropriate marker system and can generally distinguish greater diversity [22]. Hence, the LNRs and MNRs were excluded from designing locus specific primers. In our study, “TC/AG” repeats constituted the most frequent DNR microsatellite variant (25.5%) followed by “CT/GA”. While “AT/TA” and “AG/TC” repeats were reported as the most frequent in plant genomes [17,23-29]. He et al. [30] identified “GA/CT” as the most frequently occurring di-repeat motifs in groundnut. Our results revealed the presence of both the types of DNR motifs indicating a possibility that these markers would be able to distinguish greater diversity among mulberry accessions. The least abundant DNR motifs found in genomic SSRs was “CA/GT and CG/GC”. The frequency of “GC” repeats was generally less in genomic regions of most plants as reported in peach [31], coffee [32], rubber tree [33], wheat [34] and soybean [35]. While “GAA” repeats were most frequent (15.9%) among the TNRs, “AAAT” repeats were the most frequent tetra nucleotide repeats (16.6%). Similarly, “AAAAC” and “AAAAAG” repeat types were more frequent among the PNR and HNR groups, respectively.

**Table 1:** Sequences analyzed while developing genomic and genic SSR markers in mulberry

**Library**	**No. of colonies screened**	**No. of clones sequenced/Transcripts screened**	**Clones with SSR repeats**	**Sequences containing more than one SSR**	**Total no. of repeats**	**Primers developed**	**Primers standardized/Locus specific amplification**
**Genomic**	1588	1094	484	234	900	222	137
**EST**	-	2391	800	254	1155	136	51
**Total**		3485	1284	488	2055	358	188

Based on the repeat sequences, the microsatellite regions were classified as perfect, interrupted (more than one of the same repeat motif spaced by a few base pairs) and compound repeats (different repeat motifs occurring tandemly and/or interrupted by a few base pairs). Details about the genomic SSR marker types, their repeat motifs detected in the enrichment library and the gene bank accession number are presented in Table 2. Of the repeat regions identified, 74.5% were perfect, 6.5% were interrupted and 19% were compound repeats. Repeat regions of the “perfect” type are more common in plant genome compared with “interrupted” or “compound” [36,37]. Though greater representation of compound repeat motifs is not common in plant genomes, they seem to exhibit greater levels of polymorphism and hence have a distinct advantage in mapping and diversity analysis [38-41].

**Table 2:** Details of the genomic SSR markers developed for mulberry

**Sl no**	**Primer name**	**Primer sequence**	**GenBank-ID**	**Amplicon size**	**Repeat motif**	**Ta (°C)**	**Repeat type**
1	MulSSRIF	GATCTGAAGTCACCCAGCC	GF101960	236	TC	56.8	Perfect
	MulSSRIR	GCAGAATCTTTTCAGCTTCCA					
2	MulSSR2F	GGTGCCTGAAGATATGTGG	BV722881	154	AC	56.8	Perfect
	MulSSR2R	CTCTGAGGGAAGCAGAAG					
3	MulSSR23F	CGGAAACAGCCCAAAGAAGG	GF101977	223	AAACCT	56.8	Perfect
	MulSSR23R	AGGAGGGGTTTAGGGG					
4	MulSSR26F	CCACTGGTGCCTGAAG	BV722891	282	AC	56.8	Perfect
	MulSSR26R	CATCTCATACTGGGGC					
5	MulSSR-82 F	CAATCACTAACGGGGGAAG	BV722895	240	CT	56.8	Perfect
	MulSSR-82R	GCTCTTTTTGGTGCTCC					
6	MULSSR59F	GGTTTCATTTTCCCTCTCGA	BV722893	243	TTC	56.8	Perfect
	MULSSR59R	GGCCGATGCGAACAGA					
7	MULSSR85F	CCGGAGAAATTCCAAAGG	BV722896	304	TC	56.8	Perfect
	MULSSR85R	CATCCAGGCATCTGATTG					
8	MULSSR69F	CAATATTACCACCCTCAC	GF101963	294	TC	56.8	Perfect
	MULSSR69R	GAAATGGTTTGCATCC					
9	M2SSR1F	CTCTCGAGAAAGCCATCA	GF107867	217	CA	50	Perfect
	M2SSR1R	GGTTGTCAAGTAGGACCG					
10	M2SSR5F	GCTCAGATTCGGTCATGG	GF109684	186	TC	50	Perfect
	M2SSR5R	CTGCTTCATGGTATCAGAGCAAGG					
11	M2SSR12F	GCGACCATTCAACAGAACCA	GF107890	270	AG	50	Perfect
	M2SSR12R	GTGTTGTGGTTACTGGTTCC					
12	M2SSR13F	GTGTGTTGAGTGTAGCGGC	GF107891	154	GT	58	Perfect
	M2SSR13R	CGACGAAGATAACGACACGAC					
13	M2SSR19aF	GAAGAGCTCGCTACAAGG	GF107894	178	TTTTC	51.5	Perfect
	M2SSR19aR	GAAAGGCATGCTGCTCATG					
14	M2SSR20F	CTAGAGAATCTTGGGCGATCC	GF107896	230	TC	55	Perfect
	M2SSR20R	ACCGAGCGCTAGTTGTCAG					
15	M2SSR21F	GTTGCTGTGTGCTTGTGG	GF107897	247	TG	45	Perfect
	M2SSR21R	ACACAACACGTCAACCCAGA					
16	M2SSR53F	GTTGCTGAGCGTGGTGATAG	GF109658	172	AG	50	Perfect
	M2SSR53R	ACGACACGCACACACGTC					
17	M2SSR65F	GGCTGATAATCGCAATGC	GF107874	173	AGG	51.5	Perfect
	M2SSR65R	GCGTGCCCACGTAGGAAG					
18	M2SSR67F	CGAGAAATTCCGACTCCATGGTC	GF107901	158	CTC	55	Perfect
	M2SSR67R	CCGGTGGTAGTGTTGCAAGAG					
19	M2SSR68F	AATTCCGACTCCATGGTCAG	GF107902	211	TCT	51.5	Perfect
	M2SSR68F	TTCCGGTGGTAGTGTTGC					
20	M2SSR93F	ATAGCCGATTTTGCAGGC	GF107877	243	CTCC	50	Perfect
	M2SSR93R	GAAATTCCGACTCCATGGTC					
21	M2SSR94bF	ATTAGCCGTGCATCTCTGG	GF107909	295	ACTA	55	Perfect
	M2SSR94bR	CGATCACTTTCATGATCCGGG					
22	M2SSR102F	GAGCAAGGTTTCTGAACCC	GF107910	203	AAG	51.5	Perfect
	M2SSR102R	CTCAGCAGTCGTCTGAGG					
23	M2SSR121F	CGATCTGAAAGATGTCGTGC	GF107913	210	CAC	45	Perfect
	M2SSR121R	GCAACCGTCGTTCTCAGC					
24	Mul3SSR1F	CGGAAAGGGTCATGTTG	KF030980	150	AAAT	53	Perfect
	Mul3SSR1R	CTGTCGTTATTGAGAGAGCAGG					
25	Mul3SSR2F	GCTAGCAGATCCCACC	KF030981	261	CT, GAGACC	53	Perfect
	Mul3SSR2R	CAGCTCCTCTTCCACAAGC					
26	Mul3SSR4F	GGAGCAGTCAATCTCTTG	KF030982	314	(ATATAC)CAC(TA)	50	interrupted
	Mul3SSR4R	CTGGGGTTCAAACTAAGCTC					
27	Mul3SSR6F	GAGAGGTCGCCCCTTAG	KF030983	335	GT	51.5	Perfect
	Mul3SSR6R	GCCTCACAGGAGAACACC					
28	Mul3SSR7F	CCATGGCTCTTTTGGTC	KF030984	198	CTG	48.5	Perfect
	Mul3SSR7R	GCAGAATCCAGCTTTTTGG					
29	Mul3SSR9F	GACCAGCCATGAGCCTAC	KF030985	378	GT, GA	51.5	Compound
	Mul3SSR9R	GGTTCACAACCACAATCTCC					
30	Mul3SSR14F	GGCGGTTTAGGAATATAGC	KF030986	227	AG	47.5	Perfect
	Mul3SSR14R	CCAAAACGAGAAGAACG					
31	Mul3SSR16F	CTAGTAGCAGATCACCAC	KF030987	207	A, AAAAG	49.5	Compound
	Mul3SSR16R	CGGTCTCTCCCTAATCC					
32	Mul3SSR17 F	GTCTTGCACTAGGAGAGG	KF030988	345	GT	50.5	Perfect
	Mul3SSR17R	CTCACAGGAGAACACCACC					
33	Mul3SSR19F	CCAAGTCCTCCTCCAG	KF030989	172	GAA	50	Perfect
	Mul3SSR19R	GTTTTGTGACTTGCCG					
34	Mul3SSR20F	CTAGCAGATCGTGGCATTG	KF030990	252	(CT)TTCTCTAT(CT)	51	interrupted
	Mul3SSR20R	CTCCGCCCAAAATATCACAC					
35	Mul3SSR21F	CATCGCAAATAGGTGTGG	KF030991	239	TC	52.5	Perfect
	Mul3SSR21R	GGCAGTGAGAGCAAGGAG					
36	Mul3SSR23F	GCTAGCAGATCCCAAG	KF030992	224	TGCCAC, TCT	53.5	Compound
	Mul3SSR23R	CGAAACCCGCATTCATTC					
37	Mul3SSR24F	GCTCTTGTTGACACTGGC	KF030993	225	TC	51	Perfect
	Mul3SSR24R	CCGATTGTTTAAGGCC					
38	Mul3SSR25F	GAGCCTTGTTCACCAC	KF030994	155	AAG	50	Perfect
	Mul3SSR25R	GGTCAACTTTCATGCC					
39	Mul3SSR26F	GGTATGAGAGCTTCGCAC	KF030995	202	(TC)G(TC)	52	interrupted
	Mul3SSR26R	GTCTCGGGAACAACAGC					
40	Mul3SSR28F	GGATCTTGCCATCTAGTGTG	KF030996	112	TA,TG	53.5	Compound
	Mul3SSR28R	GCAGAATCATAGAGGACC					
41	Mul3SSR31F	GATCCACTTCCACTCCCAG	KF030997	382	GTC, TTC	52	Compound
	Mul3SSR31R	GGACGCATGAGGTTTTAGG					
42	Mul3SSR33F	CTCCCGGATAAAAGACAACC	KF030998	390	GAA	48.5	Perfect
	Mul3SSR33R	CCTTGCTCATCATCATCG					
43	Mul3SSR34F	CATTTTCCTCCTGACC	KF030999	221	GA	53	Perfect
	Mul3SSR34R	CAGTCCACGTCAGTTTC					
44	Mul3SSR36F	GCAGAATCCCGGAGAAGAG	KF031000	329	GAA	53	Perfect
	Mul3SSR36R	GCAGAATCCCCTGTTTGG					
45	Mul3SSR41F	CATCGCTCGTTTTCGCATC	KF031001	251	CTT	49	Perfect
	Mul3SSR41R	CACTAGCCCCTGCACC					
46	Mul3SSR43F	CTCTGGAGTACAAGAACCG	KF031002	345	GAA	49.5	Perfect
	Mul3SSR43R	GGCACGATCCCAATCAAG					
47	Mul3SSR44F	CGCGTATTTCGGATTTCC	KF031003	238	CT, CA	52	Compound
	Mul3SSR44R	GCTAGCAGAATCCCATC					
48	Mul3SSR49F	CAACATCAACACCGATCACC	KF031004	140	TCA	52	Perfect
	Mul3SSR49R	GCAGAATCCCACCAACATC					
49	Mul3SSR50F	CTAGCAGATCCACCAAACC	KF031005	161	CTT	53	Perfect
	Mul3SSR50R	GTTGTTGTACTCTCGCACG					
50	Mul3SSR52F	CAGATCCCATACACAAAGCC	KF031006	391	TTTTTC	51.5	interrupted
	Mul3SSR52R	GTGAGAGAACCCGAGAAG					
51	Mul3SSR53F	CAGCTATGACCATGATTACGCC	KF031007	124	AAAAC	50.5	Perfect
	Mul3SSR53R	GGACCCTTGATGGCATTG					
52	Mul3SSR64F	GACGAAAACCGATGAAGAGG	KC408230	380	ATGAGC	47.9	Perfect
	Mul3SSR64R	GACCGGTAAAACCACACACC					
53	Mul3SSR65F	CTGGAGTACAAGAACCGCAAC	KC408231	220	GAA	53.8	Perfect
	Mul3SSR65R	GCCCTCCACCATTGAACTAAG					
54	Mul3SSR66F	GCGAATGATGAAAACGGAGAGG	KC408232	262	TTTTA	52.8	Perfect
	Mul3SSR66R	GCGGTTAGTTGCCTAGTTGG					
55	Mul3SSR67F	ATACCACGTTCCGGTGTG	KC408233	304	GT, GA	52.8	Compound
	Mul3SSR67R	CATACCGTGCCCCAACTTAC					
56	Mul3SSR70F	GAAGAGGGGAGAGGGAGAGA	KC408236	187	AAATAA	54.1	Perfect
	Mul3SSR70R	CAACCAGGATCCAAATAGAAGC					
57	Mul3SSR71F	GGATACTACCTGTTTGGTTGCTG	KC408237	360	AAAT, GAA	54.5	Compound
	Mul3SSR71R	ATTCCCTCCTCAACGAC					
58	Mul3SSR72F	CATCCTTCGAATCCAAGAGC	KC408238	231	(AG)TTTACCCAAAGAAT(AG)	50.8	interrupted
	Mul3SSR72R	CGAGAGGAAATCCTCACAGC					
59	Mul3SSR73F	GGGGAGGTAGCTGATGTGTC	KC408239	318	TA, TATT	49.1	Compound
	Mul3SSR73R	AGCATGCCCTTCCATATCAC					
60	Mul3SSR74F	CCCATTGAGGGTTTTGTGAG	KC408240	407	AG, GTGAGC	54.8	Compound
	Mul3SSR74R	ATGTGAGCTCGGGATTTGAC					
61	Mul3SSR75F	CAGGTTGAACGCCCATTACTC	KC408241	102	CT, TCA, TC	47.9	compound
	Mul3SSR75R	GTGCAGAATGTCAGTATGCG					
62	Mul3SSR77F	ACTCCGCCTGAAGAACGAAG	KC408243	254	AGA	54.8	Perfect
	Mul3SSR77R	TAGCAGAATCCCCTGTTTGG					
63	Mul3SSR80F	GAGCCGTTTGATTTCCGTC	KC408245	158	CT	47.9	Perfect
	Mul3SSR80R	CAACGGTCGGTGAAAAAGC					
64	Mul3SSR91F	CATGAACCGTTGGATCACAG	KC408246	277	AG	54.8	Perfect
	Mul3SSR91R	ATCCCAGATCCCAAATACCC					
65	Mul3SSR93F	CAGCCAATGCACTTTTAACG	KC408248	343	AC	49.1	Perfect
	Mul3SSR93R	GTGGAGCTTCTGTTGAGC					
66	Mul3SSR94F	CCCTCATGTGTTCCATCTACC	KC408249	198	AAAACAA	52.8	perfect
	Mul3SSR94R	CAGAATCACAGCCGAGGAAG					
67	Mul3SSR95F	GATCATCGTGCCAATAAGCC	KC408250	209	AG	52.8	perfect
	Mul3SSR95R	TAAGAGCTGAGAGGGGAAGC					
68	Mul3SSR97F	TCCACCACTGAACCAAATC	KC408358	292	GAA	50.8	Perfect
	Mul3SSR97R	ATTAGGGTTGTGACGACGAC					
69	Mul3SSR98F	ACGACAATGCTGTCGTCTTG	KC408252	286	TG	55.2	Perfect
	Mul3SSR98R	CGATTCGGAAAGCAAACCAAAC					
70	Mul3SSR99F	AGGCAAAGGAGCAGGATG	KC408253	272	TTC	58.5	perfect
	Mul3SSR99R	GTGGTCACTGCAAAAAGC					
71	Mul3SSR101F	TGAGCCAAGACAAGGAGACA	KC408255	330	AC	50.8	Perfect
	Mul3SSR101R	AGCTAGCAGAATCCCCTTGA					
72	Mul3SSR102F	TTGGTTGCTGAGAAATGCAG	KC408256	230	AAAT, GAA	55.4	Compound
	Mul3SSR102R	TTGTCGATGGAAAACACGAC					
73	Mul3SSR103F	GGTCAGATCAGTTTCGTTGC	KC408257	258	AG	53.3	Perfect
	Mul3SSR103R	GTAAGAGCTGAGAGGGGAAG					
74	Mul3SSR104F	GAAGAGCCGACAAAGAATGG	KC408258	225	ATGAGC, GCAGAGAA	53.3	Compound
	Mul3SSR104R	GGAATGCTTGACCTTTGACC					
75	Mul3SSR105F	GCAGAATCCCAAGTTAATGCC	KC408259	254	TCT, TGCCAC	57.1	Compound
	Mul3SSR105R	CCTCATAGAGTACAGGAACCG					
76	Mul3SSR108F	TCTGCCATGGATGCGTGC	KC408262	215	CCTCT, TC, TC	54.1	Compound
	Mul3SSR108R	GACAGAAACCCGGCAGAAG					
77	Mul3SSR114F	GCAACTCTGCCTTGTTTTC	KC408266	106	AG	58.5	Perfect
	Mul3SSR114R	TGGTGCCTTAGACCAGAC					
78	Mul3SSR116F	GATTTTCAGCGCATGGTTC	KC408267	382	TTTTA, AATA	58.5	Compound
	Mul3SSR116R	CCAAGGAAGGTGAAATCC					
79	Mul3SSR118F	CATGAACCGTTGGATCACAG	KC408269	277	AG	53.3	Perfect
	Mul3SSR118R	ATCCCAGATCCCAAATACCC					
80	Mul3SSR122F	GGTGATGGGCTTTTGATG	KC408273	219	ATC	51.7	Perfect
	Mul3SSR122R	GTTGGATCTGAGGAGGGTC					
81	Mul3SSR124F	GGGTGCCAAGGAAAGGA	KC408275	228	TCTTTC	54.8	Perfect
	Mul3SSR124R	AGAGAGATTCGGCAAAACC					
82	Mul3SSR125F	CTTTGATGATGCTTCCTCTGC	KC408276	261	CTT, CTA	54.1	Compound
	Mul3SSR125R	GTGCACGGAATTTGCTACTG					
83	Mul3SSR126F	GGATGCTATTGCCTAAAGTG	KC408277	199	AAAAG, AAAAGA	52.8	Compound
	Mul3SSR126R	GCAGAATCAGAAGTGTTGTCC					
84	Mul3SSR127F	CGATTGCCACATGTTCAGAC	KC408278	309	AC	52.8	Perfect
	Mul3SSR127R	GGCAGACCCGATAAGCAGTA					
85	Mul3SSR131F	ACTGTGCTTCGTGGAGTTG	KC408279	305	CT, TCA	55.4	Compound
	Mul3SSR131R	GAGAGCTTCGAGAGGGAGG					
86	Mul3SSR135F	GATCATCACAAAAAGGCTGG	KC408282	137	TC	55.4	Perfect
	Mul3SSR135R	GATTGCCGACACTCGTATC					
87	Mul3SSR141F	TTGGTGCACTTGCCAAAC	KC408286	336	TTTGTT, T	52.8	Compound
	Mul3SSR141R	TCACCTCGCATAGACCAC					
88	Mul3SSR142F	GCAGAATCCCAAACTTGAGAG	KC408287	213	(AG)AAGCTGAAAATGGGGTGT(AG)	54.5	interrupted
	Mul3SSR142R	CACAGTTAGCATCACCATGTC					
89	Mul3SSR143F	TGCCACCTTCTCCAATATG	KC408288	151	TTA	54.5	Perfect
	Mul3SSR143R	CGGGAATCGGGATTAAG					
90	Mul3SSR144F	GATATGGGAACAAGGGCACTG	KC408289	284	CATCAC, ACT	54.5	Compound
	Mul3SSR144R	CTGTTTGATGAAGCCATGATG					
91	Mul3SSR145F	CCTTCTTCCCCATACCCAC	KC408290	165	TCA	50.4	perfect
	Mul3SSR145R	CATTTCGGAAGCTTGTCCA					
92	Mul3SSR146F	CAACCGATTACATGGTGTGG	KC408291	256	CT	50.4	perfect
	Mul3SSR146R	TTCCGCAGCAAGCTTTAC					
93	Mul3SSR148F	AGGCAATGACAAACGGAAG	KC408293	156	CAA	45.1	Perfect
	Mul3SSR148R	GCAACCACTTCTGTGTGAGC					
94	Mul3SSR149F	TGTCTCTTGGTCAGCGTCTC	KC408294	280	(AC)TATACATTCGT(AC)	54.8	interrupted
	Mul3SSR149R	CATTTCCCAGAAAGCCACTTC					
95	Mul3SSR150F	TCCTGTCTTAGATCGCAACG	KC408295	226	TTTTA, AAG	54.8	Compound
	Mul3SSR150R	GGTGGCAGGGATTAATGAG					
96	Mul3SSR151F	GAGTTTGCAGCCTCAGTATGG	KC408296	196	GT, T	54.8	Compound
	Mul3SSR151R	CGTGCTTGGAGTAAGGGAAG					
97	Mul3SSR152F	TCTCTGTCTGCGCATCAATC	KC408297	189	TC	54.5	Perfect
	Mul3SSR152R	GCAGAATCCCGATTTTACAG					
98	Mul3SSR153F	GGGCATTGTATTGTCCAAGC	KC408298	302	TTA	51.7	Perfect
	Mul3SSR153R	GAGTAGCCGACATAAATCAGC					
99	Mul3SSR155F	ACCCTAAATTGGGACGGAAG	KC408300	105	AAG	54.5	Perfect
	Mul3SSR155R	CGATTTCTACGAATGCCAGAC					
100	Mul3SSR156F	CCCACCCAATCACAATAACC	KC408301	190	GAA		Perfect
	Mul3SSR156R	GTCAACTCCCGAGCTCAC					
101	Mul3SSR159F	CCCAGTTGGGGTTGAGTTG	KC408304	108	TTC	51.7	Perfect
	Mul3SSR159R	CCTGTCTTGGAGAGGAGAAC					
102	Mul3SSR160F	CCCTCTCTCTCGTCGTTCTC	KC408305	171	CTT	54.8	Perfect
	Mul3SSR160R	CCCACTCAACCCGTTTTATG					
103	Mul3SSR161F	TGCATGTACTGGATGATGTG	KC408306	166	TGAAG	54.8	Perfect
	Mul3SSR161R	CTTTGGCTGTAGAAGCACG					
104	Mul3SSR163F	CAGATCTTCTCTCTTGCTCC	KC408308	221	CT, CA	54.5	Compound
	Mul3SSR163R	GTATGTTTGCTTCACGGCTC					
105	Mul3SSR164F	CGGCGGTGGAGAAACAAAG	KC408309	393	GA, AAAG, AAAAAG	54.8	Compound
	Mul3SSR164R	GTGAACCCCTGTCTTGGATG					
106	Mul3SSR166F	AAGAGAACAGTGGCCGTC	KC408311	222	ATCACC	54.8	Perfect
	Mul3SSR166R	AGGGAAAGGCAAGACTAGGG					
107	Mul3SSR167F	CCTTCTTCCCCATACCCAC	KC408312	190	TCA	49.1	Perfect
	Mul3SSR167R	CACATTTCGGAAGCTTGTCC					
108	Mul3SSR168F	CCCTTTAATCCTCTGCCTG	KC408313	267	AC	50.4	Perfect
	Mul3SSR168R	GCTGATACTTGGGGTTGG					
109	Mul3SSR169F	CCAGTTGGGGTTGAGTTGTAAC	KC408314	107	TTC	54.8	Perfect
	Mul3SSR169R	CCTGTCTTGGAGAGGAGAACC					
110	Mul3SSR170F	TAGCTAGCAGATCCCTAC	KC408315	241	GT	49.1	Perfect
	Mul3SSR170R	GGATTTCGTCGCAACCAT					
111	Mul3SSR171F	GGAGGGGTTTTCCTTGAC	KC408316	168	GAA	51.7	Perfect
	Mul3SSR171R	CGAAGTGGTGCTCTTCAC					
112	Mul3SSR172F	GCTAGGCTAAAGCCTGGAAG	KC408317	140	TGGATA	54.5	Perfect
	Mul3SSR172R	TAGTTCCGGTGACCAACTCC					
113	Mul3SSR173F	TCCCGGAACAATCTTATGG	KC408318	304	CTT, CTA	54.5	Compound
	Mul3SSR173R	CCCTAGTGCACCTTCATTTC					
114	Mul3SSR174F	AGCGGTTTCTTGTGAGCAG	KC408319	371	A, TTC	54.8	Perfect
	Mul3SSR174R	CATAGTTTGGGCCCGTTTAG					
115	Mul3SSR175F	GGAAAAGAAAGGGGGAATCAG	KC408320	127	GT	54.8	Perfect
	Mul3SSR175R	GTCTCCTTTTGGGGATACCA					
116	Mul3SSR177F	CACGTACGCAACTTTTTCC	KC408322	329	AG	49.1	Perfect
	Mul3SSR177R	GTGAGGCTTGACCTGAATG					
117	Mul3SSR178F	CAGAGGAGGATATGACATTATCAAC	KC408323	202	TC	49.1	Perfect
	Mul3SSR178R	CAAACAGAATCCCACACACG					
118	Mul3SSR179F	CCAGTTGGGGTTGAGTTGTAAC	KC408324	107	TTC	50.4	Perfect
	Mul3SSR179R	CCTGTCTTGGAGAGGAGAACC					
119	Mul3SSR180F	TCGCCACAATCTTTCACTTG	KC408325	335	TCA, TCT	54.8	Compound
	Mul3SSR180R	GCGGAGGAATTTTCCATC					
120	Mul3SSR181F	CTCTGACATTGGCAAGAAAGC	KC408326	282	TTC	51.7	Perfect
	Mul3SSR181R	GAGGAACGGCAATAAGAGG					
121	Mul3SSR183F	GATCAGGAGAGGAAGGAG	JX258829	150	AGA	52.8	Perfect
	Mul3SSR183R	CTGTCAAAACCAGCCTTG					
122	Mul3SSR184F	CATTCCTGGTGTCAGCCT	JX258830	163	(TC)T(TC)	51.7	interrupted
	Mul3SSR184R	CAGATCGGCACCAATAGT					
123	Mul3SSR185F	AGAGAGCAACCACGGGAAG	JX465665	336	AAAAAG	52.8	Perfect
	Mul3SSR185R	GTGAACCCCTGTCTTGGA					
124	Mul3SSR187F	GGACATTTCACAACCCTG	JX465667	324	AAT, CT, AGA	53.8	Compound
	Mul3SSR187R	AACTGCAAGTTGGCACAG					
125	Mul3SSR190F	AGCTGGGTGGAGGATTG	JX465669	283	AC, GCAC	54.8	Compound
	Mul3SSR190R	CCACCTCTGCAAGGATTG					
126	Mul3SSR191F	CGAATGCATAGAGGGAGAGC	JX465670	386	AAAAC	50.4	Perfect
	Mul3SSR191R	CACTTGAGGGTTCATTCAGC					
127	Mul3SSR192F	GACCTACTTCTCGAACAGTAAC	JX465671	198	AAAAC	54.8	Perfect
	Mul3SSR192R	CTTGAGGGTTCATTCAGC					
128	Mul3SSR193F	GCTAGTTCCATCGCCCATAG	JX465672	358	TTGA, TG	51.7	Compound
	Mul3SSR193R	GCATCAGATAAAGCAGGTG					
129	Mul3SSR197F	GGTGAAAGTTCGTGTGAGTCC	JX465674	186	TCT, TC	54.8	Compound
	Mul3SSR197R	TCAGCAACTAGAGTGACTTTG					
130	Mul3SSR199F	CTCAGGTACGCTGTGCTG	JX465675	238	TC	54.8	Perfect
	Mul3SSR199R	GACTCAAAGCACATGCCAAG					
131	Mul3SSR201F	CCATTGAGGGTTTTGTGAG	JX465677	406	GA, GTGAGC	54.8	Compound
	Mul3SSR201R	ATGTGAGCTCGGGATTTGAC					
132	Mul3SSR202F	CCCTCTCGATCATCACC	KC408332	230	TTC	49.1	Compound
	Mul3SSR202R	CGGAGACGTAGATGCCC					
133	Mul3SSR203F	GACCGTAGGAGAGAGTGC	KC408333	442	T, G, CG	54.8	Compound
	Mul3SSR203R	GGATACCCGCTAAACCCAC					
134	Mul3SSR205F	GCAGTTCCGAATCACGAAATAGG	KC408335	216	TTTA	49.1	Perfect
	Mul3SSR205R	CAAGGCGAGGTAAACACC					
135	Mul3SSR214F	GTGGAACAGGGAGCCAGTCT	KC408344	297	GGGCG, GAG, GAGGA	54.8	Compound
	Mul3SSR214R	CATGCACGTCTCACTCCAC					
136	Mul3SSR229F	CCTTATAGCCGATTTTGCAGGC	KC408354	247	TCT	54.8	Perfect
	Mul3SSR229R	GAAATTCCGACTCCATGGTC					
137	Mul3SSR230F	CGGGTGAGCTGGTTTGTTTC	KC408355	298	GT, TG	50.4	Compound
	Mul3SSR230R	CAGCCCCACAATCCCTACT					

### Development of genomic SSR markers

Although DNA sequences harboring microsatellite regions were captured using specific probes, primers could not be designed to all the sequences. In instances where the repeat stretch was less than 15 nucleotides or in situations where the repeat regions were close to the ends of the sequences, primers were not designed. Thus, out of the 1094 genomic clones sequenced, 222 primer pairs could be developed (Table 1). The web-based program, Primer3 (http://bioinfo.ebc.ee/mprimer3/), was adopted to design primers to the identified regions with more than 15 nucleotide repeats so as to amplify at least 150 bp fragments. The pre-cloning enrichment strategy captured specific genomic regions that were complementary to the microsatellite probes used. Thus, this approach enhanced the success of identifying specific loci that were unique in the genome. Of the set of 222 primer pairs developed, 137 (61.71%) showed locus specific amplification reiterating the advantages of the pre-cloning enrichment strategy in discovering microsatellite regions [17,30,42,43]. These locus specific markers detected 232 microsatellite motifs that could be classified into interrupted and compound repeat types (Table 2). Of these repeat types, 86 (37.1%) were DNR, 73 (31.5%) TNR, 19 (8.2%) TtNR, 27 (11.6%) PNR and 27 (11.6%) were HNR types (Figure 1). These genomic SSR markers developed for mulberry have been deposited in the NCBI GenBank database and the details of all the locus specific primers are given in Table 2.

### Isolation and characterization of genic microsatellites

A set of 2391 stress specific EST sequences obtained by subjecting K2, a leading mulberry variety [19-21], was examined for the presence of repeat motifs and 800 sequences were found to contain a total of 1155 genic microsatellite regions (Table 1). Of these, 254 sequences were found to contain more than one microsatellite locus. Mono nucleotide repeats were the most common among the sequences (Figure 1) followed by tri and hexa-repeat motifs (28.3% and 38.3% respectively). Among the factors that cause the generation of repeat sequences in the genome, replication slippage is often considered as the major mechanism. Though, this is a random phenomenon, the slippage in genic regions occurs in repeats of three bases clubbed with frame shift mutations which suppresses non-triplet repeats resulting in the abundance of TNR and HNR motifs [44-46]. A total of 180 compatible microsatellite regions were identified represented by 136 primer pairs (Figure 1). A significant 87.5% of these were perfect while 5.8% were interrupted and 6.6% were compound repeats (Table 3).

**Table 3:** Details of the genic (EST) SSR markers developed for mulberry

**Sl no**	**Primer name**	**Primer sequence**	**GenBank-ID**	**Amplicon size**	**Repeat motif**	**Ta (0°C)**	**Repeat type**
1	MESTSSR10F	CATTGCACATTGCAGGTAGC	GT629469.1	237	GTT	52.8	Perfect
	MESTSSR10R	CGGCCATCCAAAATGTTGTTC					
2	MESTSSR13F	TCTATCTCAACCGGAAGTCC	GT628644.1	230	(CAAAAG)G(AAAATA)	54.8	interrupted
	MESTSSR13R	CCAATTTGCTCGTCTTATGC					
3	MESTSSR14F	CGGCCACAGGTACTTTC	GT628768.1	202	TTGATT	50.4	Perfect
	MESTSSR14R	GGCAGCGATTTAGGATTGG					
4	MESTSSR20F	CGCAAGTGTCTCAACTG	GT629110.1	200	TGA	49.1	Perfect
	MESTSSR20R	GGAACGGATGGAGTAAG					
5	MESTSSR23F	GGCCCAAACTCCATAGC	ES448350.1	202	TAC	50.4	Perfect
	MESTSSR23R	CCGCCAATTCTAGACCAATG					
6	MESTSSR26F	CGTGATTACCTTCGGATTGG	ES448391.1	219	AGCTGG	57.9	Perfect
	MESTSSR26R	CCAACCCAGTAGACCCAGTG					
7	MESTSSR27F	CCAACATTATCCGGAACACC	ES448394.1	266	CGG	54.8	Perfect
	MESTSSR27R	GGTAAAGCCATCCGTTGC					
8	MESTSSR28F	GCCCAGTTTCCCACAGAA	ES448403.1	217	ATA	47.9	Perfect
	MESTSSR28R	GGATGGTTTGTGCGTGC					
9	MESTSSR31F	CACCAATTAAAAGCGCAGTG	ES448813.1	204	GA	57.9	Perfect
	MESTSSR31R	CTTTGTGGTTGGCTCGTG					
10	MESTSSR35F	CGTTTTCCGCTTCAGAGAG	ES448478.1	206	AG	54.8	Perfect
	MESTSSR35R	GCCGATATCCTCCTTTCCTC					
11	MESTSSR37F	CAAAAGCGGTTTGGAATAGC	ES448476.1	245	(CTTTC) CTCC(T)	54.8	interrupted
	MESTSSR37R	CCTCAACACAAAACCCACC					
12	MESTSSR40F	GAATCCTACAAGGGAGC	ES449069.1	215	AAAAT	52.8	Perfect
	MESTSSR40R	CATACAAGGATGCCCACC					
13	MESTSSR41F	GGTCGACAAGAGGTAATC	ES449022.1	121	AAAAG	56.7	Perfect
	MESTSSR41R	GAAGGCACCGAAGAGAAC					
14	MESTSSR42F	CAAGAGGTAATCCGTTC	ES448502.1	254	AG	54.8	Perfect
	MESTSSR42R	CGTTGTTAGCAGGAGC					
15	MESTSSR46F	GCCCATGTTTGCGGAG	ES449184.1	200	AG	56.7	Perfect
	MESTSSR46R	GGATTTTTCTGTCTGGGTG					
16	MESTSSR47F	GACTGCGGGAGAACAG	ES448510.1	220	CTC	54.8	Perfect
	MESTSSR47R	GTTCACCGAGGCTGAGAG					
17	MESTSSR48F	GTTGTGGTGGTTGTTGC	ES448516.1	201	TC	56.7	Perfect
	MESTSSR48R	CCTTCACTTTCTCGCC					
18	MESTSSR49F	CTTCGACGCCTTCTGCG	ES448598.1	184	GAAGA	56.7	Perfect
	MESTSSR49R	GAGCGTCTCGAAGCAGTTG					
19	MESTSSR50F	GCCGGCATGTACGGATA	ES448967.1	235	CCTAAC	54.8	Perfect
	MESTSSR50R	GTAAAAGTTTCGCCCCAGG					
20	MESTSSR51F	CCTAGGGTTTCCTTCGCTTC	ES448621.1	223	GCG	54.8	Perfect
	MESTSSR51R	CGCTTAGGCTCCTTCCTC					
21	MESTSSR52F	CTTCGTTACGCTCGCTATG	ES448640.1	261	TATTTT	56.7	Perfect
	MESTSSR52R	CCTTCTCTCAAGAATACTGG					
22	MESTSSR53F	GGCCAACATGTACGGATAG	ES449078.1	203	CCTAAC	56.7	Perfect
	MESTSSR53R	CGCCAGGTACAACAAGAAG					
23	MESTSSR56F	CATTGCGTTCCTTGAG	ES448442.1	220	ATCATG	58.8	Perfect
	MESTSSR56R	GGAGCCAAGACTCCTAAG					
24	MESTSSR59F	GAGCTCCGACGACCAC	ES448462.1	236	TCATGA	54.8	Perfect
	MESTSSR59R	GCGTCTCGACGTGAGAAATAAC					
25	MESTSSR61F	CCATAGCCTCAACGTTTC	ES448534.1	239	AAAAAC	54.8	Perfect
	MESTSSR61R	CGCTCACGTCCGTATC					
26	MESTSSR66F	GGAAAATTCATCCCCCAAGC	ES448761.1	258	TTTTTG	53.8	Perfect
	MESTSSR66R	CGATGAGAAGCTCAAGGAG					
27	MESTSSR67F	GTGCTCGTAGCTTTGATGG	ES448763.1	215	ATCGCC	54.8	Perfect
	MESTSSR67R	GCGAAGGAGAAGGAGGAGAG					
28	MESTSSR73F	CTCAAGCTATGCATCCAACGC	ES448909.1	237	CT	52.8	Perfect
	MESTSSR73R	CCACTTCGAGAGCTTCG					
29	MESTSSR74F	CCATGGCTGAGCACGAG	ES448909.1	238	GAA, GAG	52.8	compound
	MESTSSR74R	GAGCTCCAGTGTTCCTC					
30	MESTSSR76F	GATCCAGAACTCCCAAACC	ES448912.1	209	CTCCGT	50.4	Perfect
	MESTSSR76R	GGTAATCCGAGTTCGAGACG					
31	MESTSSR77F	CCATAGCCTCAACGTTTC	ES448915.1	238	AAAAAC	52.8	Perfect
	MESTSSR77R	CGCTCACGTCCGTATC					
32	MESTSSR78F	GCACTCTCAAACAAATCCTC	ES448921.1	242	AAGTGG	52.8	Perfect
	MESTSSR78R	CGTTTGGAAACGGCTACTTC					
33	MESTSSR79F	CCCATAGCCTCAACGTTTC	ES448926.1	221	AAAAAC	45.9	Perfect
	MESTSSR79R	CGACAACAACCGTCAAGTC					
34	MESTSSR85F	GTCATCTATGTCGGGTGGTC	ES448670.1	310	ATACAT	55.4	Perfect
	MESTSSR85R	CATGGAGCGTTTGTTGTGTG					
35	MESTSSR99F	GGCCAACATGTACGGATAG	ES448967.1	203	CCTAAC	50.4	Perfect
	MESTSSR99R	CGCCAGGTACAACAAGAAG					
36	MESTSSR108F	GGCTCTGAATGTCCGAGAAG	ES448289.1	246	GAGTTG	50.4	Perfect
	MESTSSR108R	GGGTGGTAGATTTGGCAC					
37	MESTSSR109F	CTCACGTCCGTATCATCG	ES448314.1	244	TTTGTT	50.4	Perfect
	MESTSSR109R	CCATTCCCATAGCCTCAAC					
38	MESTSSR111F	CATCTATGTCGGGTGGTCG	ES449122.1	299	AAAT	45.9	Perfect
	MESTSSR111R	CTATGCACAACAGGCTGC					
39	MESTSSR113F	GCCTCCCATTATGCACTATG	ES449132.1	206	AAAACA	52.8	Perfect
	MESTSSR113R	CGGATCTTCCAGGCTC					
40	MESTSSR115F	CAGGAATCAGAGCCAGAGC	ES448647.1	398	AAAAAC	53.8	Perfect
	MESTSSR115R	CTGGACCATGTGGAAGC					
41	MESTSSR117F	CATTATCCGGAACACCAGACG	ES448396.1	247	CGG	53.8	Perfect
	MESTSSR117R	GCTAAGAACCTCGCTCG					
42	MESTSSR121F	CACGTCCGTATCATCGG	ES449197.1	244	TTTGTT	52.8	Perfect
	MESTSSR121R	CCATTCCCATAGCCTCAAC					
43	MESTSSR129F	GATTACTCCAACCAACTCC	ES449040.1	223	AAAACC	52.8	Perfect
	MESTSSR129R	CAAGGGGGCTAGGAAG					
44	MESTSSR123F	CATCTATGTCGGGTGGTCG	ES448449.1	240	CT	52.8	Perfect
	MESTSSR123R	GTGTTTGCTGGACTTTGC					
45	MESTSSR126F	CACCGATGAGCCCTGGTC	ES448693.1	200	TTC	52.8	Perfect
	MESTSSR126R	GCACAATCCATCCCAAGTG					
46	MESTSSR127F	CCAACATTATCCGGAACACC	ES448594.1	285	CGG	52.8	Perfect
	MESTSSR127R	CCTGGACGGAAGAAGTGG					
47	MESTSSR131F	CCTCATTGCGTTCCTTGAG	ES448442.1	225	ATA, ATCATG	54.1	compound
	MESTSSR131R	CTGATTTGGGAGCCAAGAC					
48	MESTSSR132F	CTATGTCGGGTGGTCG	GT735086.1	473	TTTTCC	54.1	Perfect
	MESTSSR132R	CATACCGTCGGAGATGC					
49	MESTSSR136F	CCATTCCCATAGCCTC	ES449178.1	244	AAAAAC	50.5	Perfect
	MESTSSR136R	CGTCCGTATCATCGG					
50	MESTSSR134F	GGTTGTTGTCGAATCCG	ES448600.1	208	TTTGTT	55.4	Perfect
	MESTSSR134R	GTACAAACCGAACGGGAAC					
51	MESTSSR135F	CCTCATTGCGTTCCTTG	ES448442.1	219	ATCATG	54.1	Perfect
	MESTSSR135R	CCGGTGAGGTGATTGG					

It appears that the forces causing tandem repeats such as mutation, replication slippage etc., occurred more frequently in non-coding regions than the genic regions [22,45,47]. It is also possible that the lethal mutations in genic regions would subsequently eliminate the genotype while the sequence variations in non-coding regions of the genome would persist, resulting in the observation of higher frequency of sequence variations in the non-coding genomic regions. Accordingly, more numbers of repeat regions were found on the genomic regions (82%) while 48% were found in the genic regions. A large number of clones with more than 15bp of repeat motifs were found among the markers developed. Results revealed that the frequency of such markers was more in the non-coding regions of the mulberry genome than the genic regions [25]. The presence of longer repeats in the genome may have an evolutionary advantage leading to differences in the ability to adapt to new environments [48,49].

### Validation of genomic and genic SSR markers

The genic and genomic SSR markers were validated using four contrasting genotypes of *Morus alba* that were chosen based on variations in certain physiological traits [50] and seven different mulberry species (all belonging to the genus *Morus*) (Table 4). Of the 222 genomic and 136 genic SSR markers screened, 137 (62%) genomic and 51 (37%) genic SSR markers showed single locus amplification in all the *Morus* species as well as genotypes of *Morus alba* (Table 5). Further, genomic SSRs exhibited greater levels of polymorphism compared with the genic SSR markers. Such phenomenon has also been reported in other plant species [51]. Of the 188 markers examined, 87 (46.2%) detected heterozygosity in the mulberry genotypes and species with a maximum of 1.00 for markers MulSSR39, Mul3SSR26 Mul3SSR91 and Mul3SSR135, (Additional file 1). Around 41% of the genic markers also detected heterozygosity among the mulberry genotypes and species (Additional file 1). SSR markers are highly suited for mapping even in cross pollinated species because of their ability to detect heterozygosity. The markers developed in this study also detected significant levels of heterozygosity in mulberry species and genotypes.

**Table 4:** Various mulberry species (A), mulberry genotypes (B) and other related species (C) for characterizing SSR markers

**S.No**	**Genotypes**	**Family**	**Origin**	**Ploidy**	
**1**	*M. alba*	*Moraceae*	Japan	2n = 28	A
**2**	*M. assambola*	*Moraceae*	-	-	
**3**	*M. exotica*	*Moraceae*	Zimbabwe	-	
**4**	*M. indica*	*Moraceae*	India	2n = 28	
**5**	*M. lavigata*	*Moraceae*	India	2n = 3× = 42	
**6**	*M. macroura*	*Moraceae*	-	-	
**7**	*M. multicaulis*	*Moraceae*	China	2n = 28	
**8**	Dudia white	*Moraceae*	India	-	B
**9**	Himachal Local	*Moraceae*	India	-	
**10**	MS3	*Moraceae*	India	-	
**11**	UP105	*Moraceae*	India	-	
**12**	*Artocarpus heterophyllus* (Jackfruit)	*Moraceae*	Asia	2n = 56	C
**13**	*Ficus bengalensis (*Banyan)	*Moraceae*	South Asia	-	
**14**	*Ficus carica* (Fig)	*Moraceae*	South Asia	2n = 26	

(Note: All species belong to family *Moraceae*).

**Table 5:** Markers developed for mulberry and their transferability to related species

**SSR type**	**Locus specific**	**Monomorpic in *Morus *** **spp**	**Monomorpic in all species**	**Polymorphic in *Morus *** **spp**	**Primers transferable to other species**	**Transferability**		
						**Jackfruit**	**Ficus**	**Fig**
**Genomic**	**137**	**12**	**1**	**125** (91.24%)	**107** (78.10%)	**96** (70.07%)	**64** (46.71%)	**64** (46.71%)
**Genic**	**51**	**12**	**6**	**39** (76.47%)	**42** (82.35%)	**39** (76.47%)	**21** (41.17%)	**22** (43.13%)
**Total**	**188**	**24**	**7**	**164** (87.23%)	**149** (79.25%)	**135** (71.80%)	**85** (45.21%)	**86** (45.74%)

**Figure 2:**
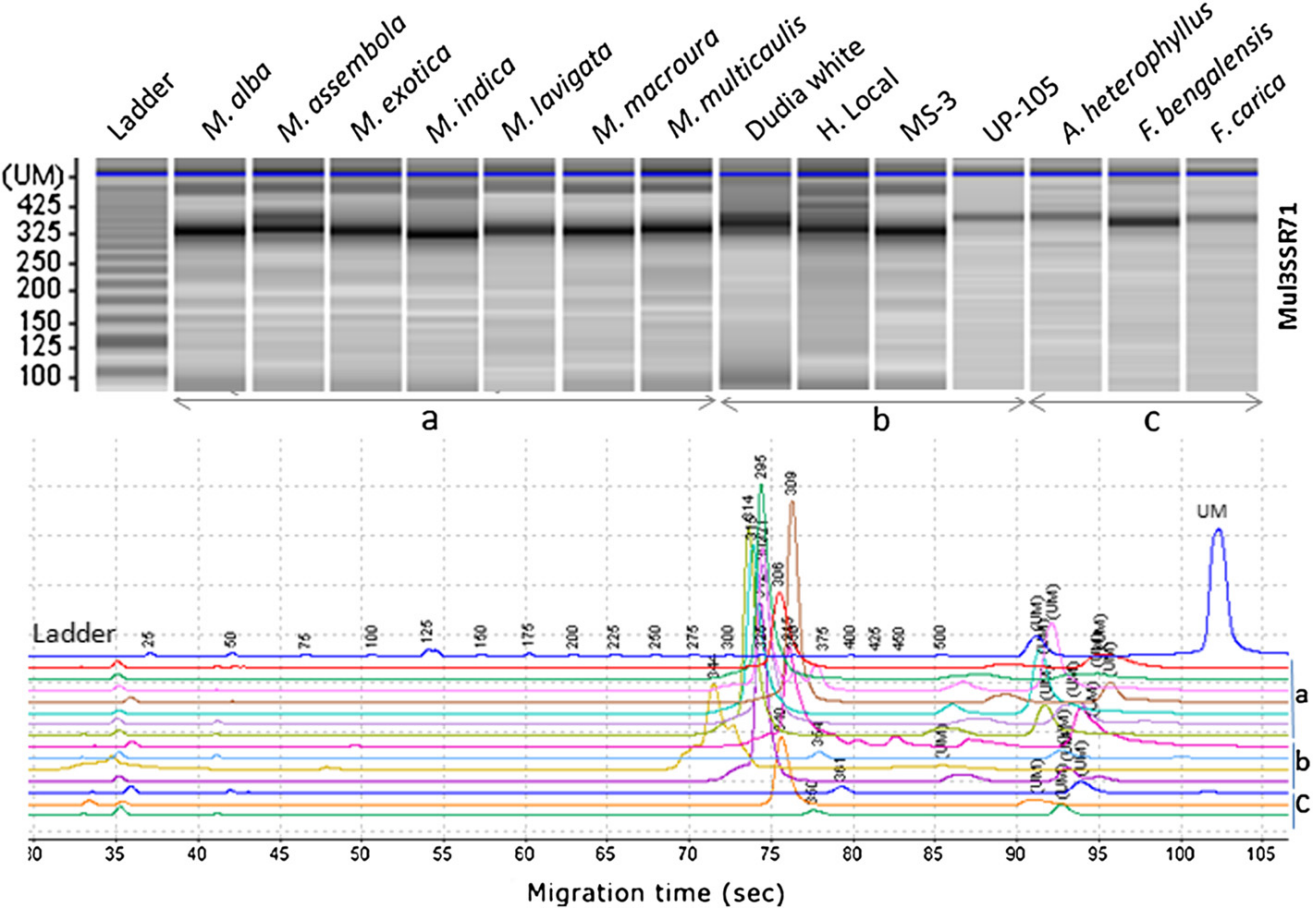
**Gel image generated by the MultiNA for different Mulberry species, genotypes and other related species.** All species and genotypes belong to family *Moraceae*. (a) Morus species, (b) Mulberry genotypes and (c) other related species.

Variations in the genic regions, though less frequent, would have a greater possibility of having a direct role in altering the phenotype of an organism [52]. The variability obtained for the SSR markers across mulberry species and genotypes was analyzed using Power Marker version 3.25 and the results are summarized in Table 6. A total of 936 alleles were obtained from 188 markers of which 164 (87%) were polymorphic among the mulberry species and genotypes. These markers revealed an allelic diversity ranging from 1 to 17 with an average of 4.97 alleles per marker locus (Figure 2/Table 6). Earlier reports on allelic diversity of mulberry SSR markers had revealed an average of 4.9 [18], 5.1 [53] and 18.6 [17] alleles per locus. This allelic diversity can be effectively used for various applications ranging from diversity, evolutionary history and QTL mapping of complex traits in mulberry.

**Table 6:** Genetic diversity and polymorphic information revealed by markers developed in mulberry and related species

**Samples**	**Range**	**Genetic diversity**	**No. of alleles**	**Heterozygosity**	**PIC**
**All species and genotypes**	**Min**	0.0799	2	0.000	0.0767
	**Max**	0.9464	22	0.9091	0.9438
	**Mean**	0.5969	5.47	0.1830	0.5592
***Morus *** **species only**	**Min**	0.0000	1	0.000	0.0000
	**Max**	0.9339	17	1.0000	0.9299
	**Mean**	0.5860	4.97	0.1881	0.5431
**Other related species**	**Min**	0.0000	2	0.0000	0.0000
	**Max**	0.8333	6	1.0000	0.8102
	**Mean**	0.4090	2.57	0.0532	0.3457

While most of the markers developed in the study amplified the genomic DNA of all mulberry species and genotypes, a few also included private or rare alleles. For instance, Mul3SSR153 only could amplify a few particular mulberry species (*M. lavigata*, *M. assambola)* and a mulberry genotype (Dudia white). Such private/rare alleles have great utility in establishing the genetic authenticity of a particular species and/or genotype in germplasm characterization as well as in genetic screening experiments [54].

Most of the genic and genomic SSR markers developed in this study were highly informative with an average PIC value of 0.543 which ranged from 0.000 to 0.929 among mulberry species and genotypes (Table 6). Percentage of variation explained by the principal component analysis also revealed that 41% of the markers were effective in discriminating the variation among the mulberry species and genotypes confirming their efficiency in detecting genetic variations even among closely related varieties.

Two mulberry genotypes viz., Dudia white and UP105 were identified as contrasting lines differing in root traits and WUE in earlier studies [50]. These lines were crossed and a F_1_ segregating population was developed. Of the 188 markers examined, 94 genomic and 22 genic markers were found to be polymorphic between these two parents. These polymorphic markers would be a very useful genomic resource for constructing a genetic linkage map for mulberry. This work is in progress and when done would lead to the determination of the linkage between markers and their position on mulberry linkage groups.

In the present investigation, we report a large number of genic and genomic SSR markers that can be exploited to examine the diversity among mulberry genotypes and species. However, the relevance of the marker system would increase if they are transferable to other species.

### Transferability of the SSR markers to other related species

The transferability of the mulberry SSR markers was examined using three species belonging to the family *Moraceae viz.,*Ficus (*F.bengalensis*), Fig (*F. carica*), and Jackfruit (*A. heterophyllus*) (Table 4). Of all the markers evaluated 78% (107) genomic and 82% genic (42) markers showed locus specific amplification in at least one of the three species studied (Table 5). Around 30% of the markers were transferable to all the three species. Of the 107 genomic and 42 genic markers, 70% and 76% were transferable to jackfruit. The transferability of these markers was relatively low in Fig and Ficus, which ranged between 41 to 46% (Table 5). It can be perceived that the genic regions of related genomes would be more conserved than the non-coding regions and hence would have higher transferability [55]. These markers would be highly useful for genome mapping and comparative genomics in mulberry and other closely related species belonging to *Moraceae*.

Several reports confirm the molecular relatedness of mulberry with a few other plant species belonging to the family *Moraceae*[56,57]. Thus, the effective transferability of both genic and genomic SSR markers to these species can be expected. In this context, the present study is significant as a large proportion of the mulberry markers were found to be effectively transferable to these closely related species of family *Moraceae*.

### Diversity analysis

**Figure 3:**
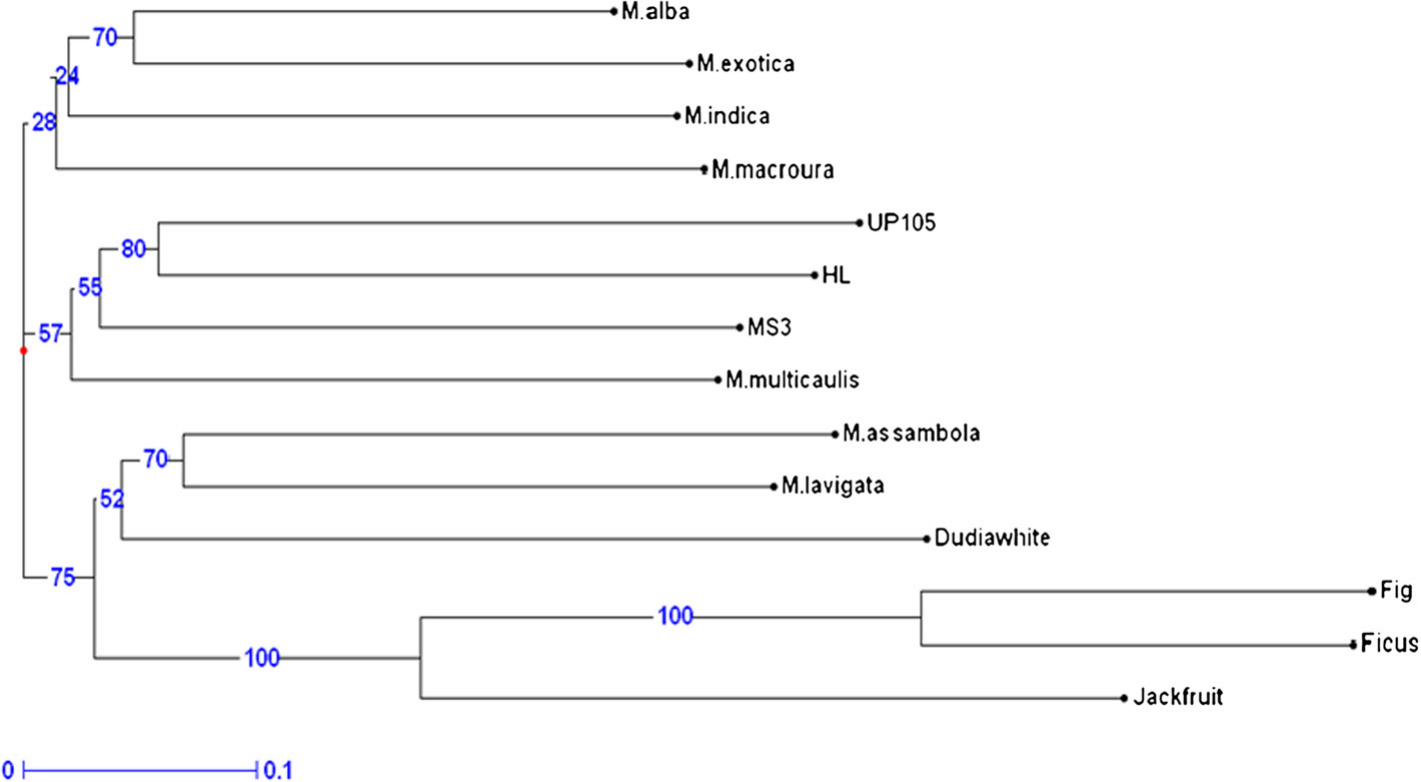
**Genetic diversity analysis of mulberry species, genotypes and three related species using both genomic and genic microsatellite markers.** Ficus (*Ficus bengalensis*), Fig (*Ficus carica*) and Jackfruit (*Artocarpus heterophyllus*) were the closely related species examined for the transferability of microsatellite markers developed. All species and genotypes belong to family *Moraceae.*

**Figure 4:**
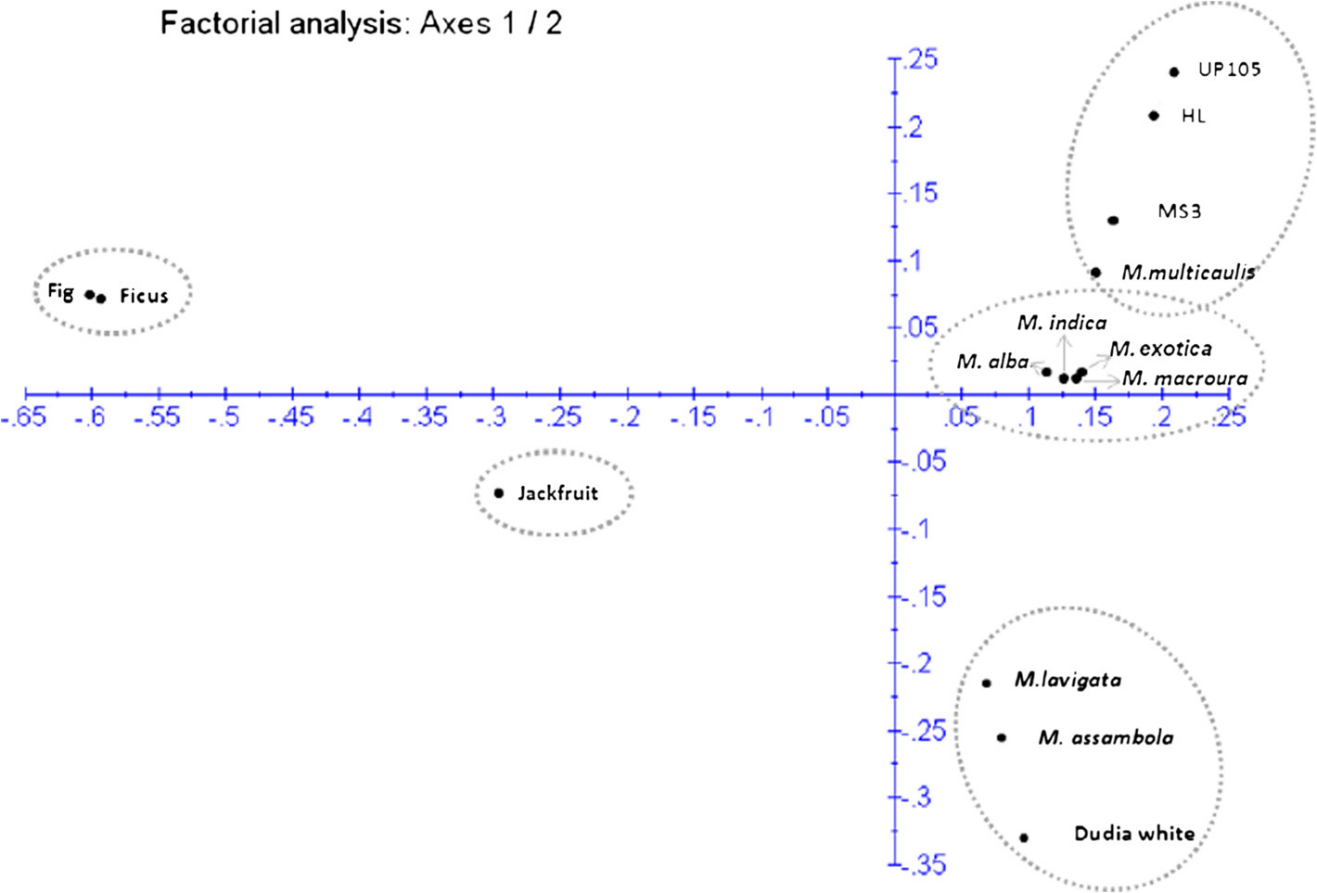
**Factorial analysis for grouping of mulberry species, genotypes and three related species using genomic and genic SSR markers.** Ficus (*Ficus bengalensis*), Fig (*Ficus carica*) and Jackfruit (*Artocarpus heterophyllus*) were the closely related species examined for the transferability of microsatellite markers developed. All species and genotypes belong to family *Moraceae.*

Genetic diversity among the mulberry and three closely related species from the family *Moraceae* was analyzed using the 188 locus specific markers. We used two clustering algorithms viz., Unweighted Neighbor Joining (NJ) and factorial analysis (FA) to group the species and genotypes. The results of genetic relationships among the species and mulberry genotypes based on NJ and FA is presented in Figures 3 and 4. Both the algorithms were congruent and grouped the species and genotypes into four clusters. *A. heterophyllus*, *F. bengalensis* and *F. carica* segregated into a distinct cluster (I) while other mulberry species and genotypes clustered separately (II, III and IV). It was interesting to note that Dudia white clustered along with *M. lavigata* and *M. assambola,* while all other mulberry species and genotypes grouped into clusters III and IV. Though the dendrogram in Figure 3 indicates clusters III and IV as different, based on the boot strap values, these clusters could be considered as not significantly distinct. Therefore it is apparent that all the mulberry genotypes and species share common alleles except the genotype Dudia white and mulberry species *M. lavigata* and *M. assambola.*The diversity structure represented by the factorial analysis also indicated a similar grouping pattern for the mulberry species and genotypes (Figure 4). Though Dudia white is often considered as a genotype of *M. alba*, there is no firm molecular evidence for its origin.

The genetic relatedness of the 14 species and genotypes is explained in the Table 7. Based on the dissimilarity matrix Fig and UP105 showed maximum dissimilarity (93.8%) and Fig and Ficus showed the least (38%). Among the mulberry species and genotypes, the minimum genetic dissimilarity (44.4%) was observed between *M. alba* and *M. exotica* and highest dissimilarity of 74.7% was found between Dudia white and UP105. These two genotypes significantly differed in physiological traits such as root length and water use efficiency [50].

**Table 7:** Dissimilarity matrix of mulberry and other related species tested for transferability of genic and genomic SSR markers

**Accessions**	**Mulberry species**							**Mulberry genotypes**				**Related species**		
	***M. Lavigata***	***M. indica***	***M. assambola***	***M. macroura***	***M. multicaulis***	***M. exotica***	***M. alba***	**Himachal Local**	**UP105**	**Dudia white**	**MS3**	**Jackfruit**	**Ficus**	**Fig**
***M. lavigata***	1													
***M.indica***	0.602	1												
***M.assambola***	0.535	0.629	1											
***M.macroura***	0.615	0.544	0.641	1										
***M.multicaulis***	0.620	0.578	0.647	0.590	1									
***M.exotica***	0.608	0.527	0.634	0.550	0.584	1								
***M.alba***	0.576	0.495	0.602	0.518	0.552	0.444	1							
**Himachal local**	0.662	0.620	0.689	0.632	0.597	0.626	0.594	1						
**UP105**	0.682	0.640	0.708	0.652	0.616	0.645	0.613	0.582	1					
**Dudia white**	0.625	0.668	0.651	0.680	0.686	0.673	0.641	0.728	0.747	1				
**MS3**	0.630	0.587	0.656	0.600	0.564	0.593	0.561	0.581	0.600	0.695	1			
**Jackfruit**	0.734	0.753	0.760	0.765	0.771	0.758	0.727	0.813	0.832	0.799	0.780	1		
**Ficus**	0.833	0.852	0.859	0.864	0.870	0.857	0.825	0.912	0.931	0.898	0.879	0.704	1	
**Fig**	0.840	0.859	0.867	0.871	0.877	0.865	0.833	0.919	0.938	0.905	0.886	0.711	0.380	1

Overall, the diversity analysis clearly indicates that the markers reported in this study are very well conserved across the taxa and can be effectively utilized to study the genetic relationship among varieties, genotypes and species of *Moraceae*.

## Conclusion

Considering the commercial importance of mulberry and the complexity of trait based breeding, a focused molecular breeding strategy needs to be evolved for the genetic enhancement of this crop. Lack of sufficient genomic resources such as SSR markers has been one of the major constraints. We report a total of 188 robust locus specific SSR markers generated by analyzing 3485 genic and genomic sequences of mulberry genome. The markers developed were highly efficient in characterizing seven different mulberry species and four contrasting genotypes of *Morus alba* L. These markers also exhibited extensive transferability to other related species belonging to the family *Moraceae* viz., Ficus (*Ficus bengalensis*), Fig (*Ficus carica*) and Jackfruit (*Artocarpus heterophyllus*)*.* The markers displayed high levels of polymorphic information content (PIC) and heterozygosity, enhancing the opportunities of using these markers in diversity analysis as well as for tagging QTLs governing complex agronomic and physiological traits. All the markers developed have been deposited in NCBI/EMBL database and are publicly available.

## Methods

### Plant materials and DNA extraction

Two strategies were adopted for the generation of genomic resources of microsatellite markers for mulberry. Microsatellite motifs in the genomic regions were identified by adopting the pre-cloning enrichment strategy using the genomic DNA isolated from a mulberry genotype Dudia white. Similarly, a stress expressed sequence tag (EST) was analyzed to identify microsatellite motifs in genic regions of mulberry genome. Details of the methodology adopted are described below.

### Pre-cloning enrichment strategy for the construction of genomic library and mining of microsatellite motifs

The SSR enriched genomic library was constructed by a modified method of Saghaimaroof et al. [58]. Four micrograms of high quality genomic DNA was extracted from a genotype, Dudia white. This genotype was identified based on the extensive phenotyping carried out with a diverse set of mulberry germplasm [50]. The genomic DNA was digested by blunt-end generating restriction endonuclease, *Rsa*I (MBI Fermentas, USA). This restriction reaction generated a large number of approximately 500–1000 base pair fragments. The ligation of Super SNX linkers, consisting of a Super SNX 24-mer (5’-GTTT AAGGCCTAGCTAGCAGAATC-3’) and a phosphorylated 28-mer (5’- pGATTCTGCTAGCTAGGCCTTAAACAAAA-3’) to the blunt termini of restriction fragments was performed for 2 hours at 37°C. To ensure linker ligation, 10 μl of digested and ligated product was pre-amplified using 1.5 μl of Super SNX24 Forward primer (10 μM), 150 μM of dNTPs, 2 mM MgCl_2_, 1 unit of *Taq* DNA polymerase and 25 μg/ ml of BSA in a volume of 25 μl. Self-ligation of the linkers was avoided by adding 1 unit of the restriction enzyme, *Xmn*I. PCR amplification was carried out with a program consisting ofan initial DNA denaturation step of 95°C for 2 min followed by 20 cycles of: DNA denaturation step at 95°C for 20 s, primer annealing cycle with the appropriate temperature for specific primer pairs for 20s and a DNA extension cycle of 72°C for 2 mins. A final elongation step of 72°C for 10 min was performed to ensure complete amplification of the fragments. All PCR amplifications were carried out using an Eppendorf Master Cycler (Eppendorf, Hamburg). An aliquot of the amplicons was resolved on a 1.2% agarose gel to check the success of linker ligation.

The restriction digested and linker-ligated DNA fragments were captured by hybridizing with biotinylated microsatellite oligonucleotides (Sigma Aldrich): [CA]_17_, [AG]_16_, [AGC]_8_, [AGG]_8_, [ACGC]_5_, [ACCT]_8_, [AAC]_14_, [ATC]_14_ and [AAG]_14_. The enrichment of microsatellites was carried out in 50 μL reaction volume containing 25 μL 2× hybridization solution (12× Sodium saline citrate, 0.2% SDS), 10 μL equimolar biotinylated microsatellite oligos and 2 μg of linker ligated DNA. The hybridization of the microsatellite harboring genomic DNA fragments with the biotinylated microsatellite probes was facilitated by a touchdown temperature PCR consisting of 99 cycles of 95°C/5 min, 70°C/5 sec, 68.8°C/5 sec, 68.6°C/5 sec with step down of 0.2°C for every 5 sec until it reaches 50°C. The temperature in the tubes was then maintained at 50°C for 10 min. Subsequently, a program consisting of 20 cycles of 49.5°C/5 sec with step down of 0.5°C every 5 sec until it reaches 40°C/5 sec and finally held at 15°C.

The touchdown PCR conditions facilitate the microsatellite probes to hybridize with complimentary DNA repeat fragments (i.e., expectantly long prefect repeats) when the reaction mixture is at or near the microsatellite probes melting temperature. Hybridized fragments were selectively isolated using Streptavidin coated paramagnetic beads (Roche, Mannheim, Germany). Enriched DNA fragments were amplified with super SNX24 primers and purified using PCR purification column (Sigma, USA). The purified enriched products were ligated to pTZ57R/T vector (MBI Fermentas, USA) using T4-DNA ligase overnight at 16°C. The ligated genomic inserts were cloned in competent *E. coli DH5α* host cells and grown over night at 37°C. The transformed colonies were confirmed by performing PCR using M13 universal primers (3 μM), 100 μM dNTPs, 2 mM MgCl_2,_ 1 U Taq DNA polymerase and 1X PCR buffer, at an annealing temperature of 58°C for 30 cycles. PCR products of the recombinant clones were purified using PCR-purification column (Sigma, USA) and sequenced using M13 forward and reverse primers on ABI 3700 sequencer.

### Development of EST library to identify genic microsatellite markers

A stress transcriptome was developed by extracting the total mRNA from the leaves of water stressed and well watered mulberry plants. A widely adopted mulberry variety, K2 was used for this purpose. A modified guanidiumisothiocyanate protocol [59] was adopted to isolate total RNA from mature leaf tissue. Total messenger RNA (mRNA) was then isolated from 1 mg of total RNA using mRNA isolation kit (Promega). The mRNA was reverse transcribed to develop cDNA and the ESTs have been isolated [19]. These EST sequences were used in this investigation to develop genic SSR markers.

### SSR marker development

Initially, the sequences were analyzed to identify unique and non-redundant libraries of genic and genomic regions for designing primers. The nucleotide sequences were analyzed using the Clustal-W, an on-line toolto determine the complemetarity between pairs of sequences. The non-redundant sequences were analyzed with “Mreps” software (http://bioinfo.lifl.fr/mreps/mreps.php) to identify sequences containing microsatellite motifs. The analysis revealed the presence of a single nucleotide base being the repeat motif (mono nucleotide repeat – MNR) to as high as regions with more than six bases (long nucleotide repeat – LNR). The MNR and LNR sequences were omitted from further analysis and primers were designed only the sequences with repeat motifs of two nucleotides (di-nucleotide repeats – DNR) and six nucleotides (hexa-nucleotide repeats – HNR). Primer3, also online software was used for designing appropriate primers [60]. The quality of primers was determined using the FAST PCR program and only those primers that would amplify a fragment in the range of 150 and 450 base pairs of template DNA were selected. Synthesis of these primers was outsourced to Bioserve India Pvt. Ltd., Hyderabad). Each of the primer pairs was standardized for their locus specific amplification using the genomic DNA of Dudia white as a template. Gradient-PCR was carried out in a total volume of 15 μL containing 2 ng of DNA template, 1× Taq buffer, 2 mM MgCl_2_, 0.2 mM dNTPs, 1 U Taq DNA polymerase (MBI Fermentas, USA) and 3 μM each of forward and reverse primers. Amplification was performed in a epGradient Master cycler (Eppendorf, Hamburg)with the following PCR conditions: DNA denaturation at 95°C for 5 min followed by 30 cycles of 95°C for 1 min, primer annealing temperatures ranging between 45-65°C for 45 s (depending on the Ta for each primer pair) and a DNA extension step of 72°C for 45 s and a final extension step at 72°C for 8 min. The details of the primer sequences, their annealing temperatures, expected amplicon size etc. are summarized in Table 2 and Table 3. The amplified products were resolved on 3% agarose gels. Only those primer pairs that produced unambiguous single band amplification alone were considered for the development of SSR markers in mulberry. This stringency ensured the development of robust SSR markers in mulberry which can be effectively used for diversity analysis as well as for constructing genetic linkage maps. Only such markers were further used for validation.

### Validation of markers

Each of the markers was examined for their ability in amplifying the genomic DNA from other mulberry species and genotypes. Genomic DNA was extracted from seven distinct mulberry species and four contrasting genotypes of mulberry using a modified CTAB method [61]. These four genotypes were selected based on the extensive phenotyping of a set of 295 germplasm accessions for the variability in root traits and water use efficiency. Thus, the four genotypes represent contrast for these highly relevant drought adaptive traits. The list of the mulberry species and genotypes are given in Table 4. The template DNA from the different mulberry species and genotypes were amplified using each of the primers for genic and genomic microsatellite markers. The PCR conditions followed are same as that adopted for gradient PCR, explained above. All the amplified products were analyzed on microchip based electrophoresis system MultiNA (Shimadzu biotech, Japan) and the highest peak detected by the fragment analyzer was scored for the presence of the expected band for each primer pair. The polymorphism data was scored and used for the determination of polymorphic information content (PIC) for each marker as per Liu and Muse [62], Observed heterozygosity and allele diversity were computed using the Power Marker 3.25 software [62]. The most appropriate locus specific marker competent to divulge the variation among the species and genotypes was determined by principle component analysis (PCA).

### Genetic diversity and cross species transferability

It is well known that there would be significant levels of sequence homology between closely related species and hence, there would be a possibility of a specific SSR marker detecting a similar locus in other related species. Establishment of the transferability of markers to other related species is therefore important while developing locus specific marker systems. The transferability of these markers was examined in three closely related species belonging to the family *Moraceae, namely* Ficus (*F. bengalensis*), Fig (*F. carica*) and Jackfruit (*A. heterophyllus*) (Table 4).

The percentage of transferability of the markers was calculated for each species by determining the presence of target loci to the total number of loci analyzed. The allelic diversity data obtained for all the microsatellite loci amplified were used to compute the genetic dissimilarity using DARwin v.5.0 program [63]. The dissimilarity matrix was further used to group the species according to their genetic relatedness based on Unweighted Neighbor Joining method and factorial analysis.

## Competing interests

The authors declare that they have no competing interest.

## Supplementary Material

Additional file 1 Marker-wise details of the gene diversity, heterozygosity and PIC values tested using mulberry species and genotypes. Click here for file
